# Association of Arkansas’s section 1115 Medicaid waiver with health insurance coverage

**DOI:** 10.1371/journal.pone.0231417

**Published:** 2020-04-09

**Authors:** Jim P. Stimpson, Sungchul Park, Fernando A. Wilson

**Affiliations:** 1 Dornsife School of Public Health, Drexel University, Philadelphia, Pennsylvania, United States of America; 2 Matheson Center for Health Care Studies, University of Utah, Salt Lake City, Utah, United States of America; Keck School of Medicine of the University of Southern California, UNITED STATES

## Abstract

**Objective:**

Evaluate how the use of a Section 1115 waiver in Arkansas was associated with health insurance coverage compared to Medicaid expansion states that did not use a waiver.

**Methods:**

Difference in difference analysis was conducted of 1,320,790 adults aged 19–64 with family incomes at or below 138% of the federal poverty level from the 2010–2017 American Community Survey. Arkansas was compared to states that expanded without a waiver in calendar year 2014. States that expanded Medicaid with an approved Section 1115 waiver during the study period or expanded without a waiver after 2014 or did not expand Medicaid were excluded from the analysis. The outcome measures were no health insurance coverage, Medicaid coverage, employer sponsored private insurance, and non-group direct purchase private insurance.

**Results:**

Arkansas’s use of a waiver to expand Medicaid was associated with a lower uninsured rate (-3.7%, p< 0.001), a higher Medicaid coverage rate (2.0%, p< 0.001), and a higher non-group, direct purchase private insurance coverage rate (2.9%, p< 0.001) compared to states that expanded Medicaid in 2014 without a waiver.

**Conclusion:**

Compared to states that implemented traditional Medicaid expansion, we found that Arkansas’s waiver was associated with increases in health insurance coverage rates.

## Introduction

Section 1115 waivers have the potential to foster innovative approaches to addressing access to healthcare among low-income populations by granting states increased autonomy in how they manage their Medicaid programs [1]. However, policymakers have limited evaluation data on waivers to make informed policy decisions [1–3]. States’ Section 1115 waiver programs vary substantially in structure, and thus it is important to evaluate the effect of these waivers for each individual state [1–2]. The state of Arkansas expanded Medicaid on January 1, 2014 using a Section 1115 waiver approved by the Centers for Medicare and Medicaid Services (CMS) [4]. The Section 1115 waiver in Arkansas was the first in the country to allow a state to use funds from Medicaid to pay for private insurance coverage for eligible residents. The waiver was later renamed “Arkansas Works” in 2016 and added provisions to charge beneficiaries’ premiums to establish health savings accounts [4]. Given the tumult in Section 1115 waiver approvals and judicial action, future study is required to evaluate the impact of waivers on health insurance coverage. The objective of this study is to evaluate how the use of a Section 1115 waiver in Arkansas was associated with health insurance coverage compared to Medicaid expansion states that did not use a waiver.

## Methods

This paper was considered non-human subjects research by the Drexel University review board (Protocol # 1906007226). This cross-sectional study included 1,320,790 adults aged 19–64 with family incomes at or below 138% of the federal poverty level. States that expanded without a waiver in calendar year 2014 were included in the analysis (California, Colorado, Connecticut, Delaware, DC, Hawaii, Illinois, Iowa, Kentucky, Maryland, Massachusetts, Minnesota, Nevada, New Jersey, New Mexico, New York, North Dakota, Ohio, Oregon, Rhode Island, Vermont, Washington, West Virginia). States that expanded Medicaid with an approved Section 1115 waiver during the study period (Arizona, Iowa, Indiana, Michigan, Montana, and New Hampshire) or expanded without a waiver after 2014 (Alaska, Pennsylvania, Louisiana) or did not expand Medicaid were excluded from the analysis. We used the 1-year estimates from American Community Survey (ACS) data provided by the Integrated Public Use Microdata Series (IPUMS) [5]. The date range for the ACS data used was January 1, 2010 to December 31, 2017. State decisions on Medicaid expansion and Section 1115 waivers were linked to IPUMS using a the state Federal Information Processing Standards (FIPS) code.

Consistent with other studies examining the effect of the Affordable Care Act (ACA) on health insurance coverage, we used a differences-in-differences (DD) linear probability regression to compare the association of health insurance coverage rates in Arkansas compared to what these rates would have been if Arkansas had expanded Medicaid without the waiver [6]. The state of Arkansas was defined as the treatment group. States that expanded Medicaid without a waiver were defined as the control group. Time was defined as either as pre-ACA (January 1, 2010 –December 31, 2013) or post-ACA (January 1, 2014 –December 31, 2017). The outcome variables were whether the respondent had coverage from Medicaid, private insurance (employer-sponsored or non-group, direct purchase), or did not have health insurance coverage. Control variables included age in years, sex (male, female), number of children (0,1,2,3+), marital status (married, not married), poverty (0–99%, 100–138% federal poverty level), race/ethnicity (White non-Hispanic, Hispanic, Black non-Hispanic, other race/ethnicity), education (less than high school, high school, some college, college), employment status (employed, not employed), immigration status (US native, naturalized citizen, non-citizen) [7], and residence in a metropolitan area (live in a metro area, live in a non-metro area). All analyses were conducted using Stata/MP version 15, and accounted for survey weights and adjusted for state and year fixed effects [8]. Statistical significance was assumed at 2-sided p< 0.05.

## Results

The estimates in Table 1 indicate that Arkansas’s use of a waiver to expand Medicaid was associated with a lower uninsured rate (-3.7%, p< 0.001), a higher Medicaid coverage rate (2.0%, p< 0.001), and a higher non-group, direct purchase private insurance coverage rate (2.9%, p< 0.001) compared to states that expanded Medicaid in 2014 without a waiver. There was not a statistically significant association between Arkansas’ waiver and employer-sponsored private insurance coverage rate (-0.4%, p = 0.431). The parallel trends assumption for the differences-in-differences analysis, calculated using a joint F-test across the coefficients for the pre-expansion period (2010–2013), was met through visual inspection of the trend graph and the regression estimator (F = 0.79, p = 0.497).

**Table 1:** Differences-in-differences multivariate regression model for health insurance coverage rates in Arkansas compared to states that expanded medicaid in 2014 without a Section 1115 waiver among 1,392,045 medicaid eligible adults, American Community Survey 2010–17^a^.

	Uninsured, %	Medicaid, %	Non-Group, Direct Purchase Private Insurance, %	Employer Sponsored Private Insurance, %
**Before Medicaid Expansion (2010–2013)**				
Control ^b^ –Medicaid expansion states without a Section 1115 waiver	26.5	46.5	7.2	23.8
Treated–state of Arkansas	50.1	12.6	8.6	28.1
Treatment vs. control difference	23.6 ^c^	-33.9 ^c^	1.4 ^d^	4.3^c^
**After Medicaid Expansion (2014–2017)**				
Control–Medicaid expansion states without a Section 1115 waiver	5.3	67.5	7.9	24.2
Treated–state of Arkansas	25.2	35.5	12.1	28.0
Treatment vs. control difference	19.9 ^c^	-31.9 ^c^	4.3 ^c^	3.9^c^
**Difference-in-Difference**				
After (2014–2017)–before (2010–2013) difference	-3.7 ^c^	2.0 ^c^	2.9 ^c^	-0.4^e^
R^2^	0.15	0.14	0.05	0.08

^a^ Linear probability percentage point estimates are based on the sample weights provided by the Census Bureau. Medicaid eligibility was defined by age 19–64 and income below 139% of the federal poverty level. Analyses exclude states that did not expand Medicaid and states that expanded Medicaid using a waiver and states that expanded Medicaid without a waiver after 2014. All years are calendar years (January 1 to December 31). Multivariate adjustment included the following control variables: age, sex, number of children, marital status, race/ethnicity, immigration status, poverty status, education, employment status, and metropolitan residence.

^b^ States that expanded Medicaid in calendar year 2014 without a Section 1115 waiver were defined as the control group, and the state of Arkansas was defined as the treatment group.

^c^ p < 0.001

^d^ p = 0.014

^e^ p = 0.431

## Discussion

Compared to states that implemented traditional Medicaid expansion, we found that Arkansas’s waiver was associated with increases in health insurance coverage rates. This finding is in contrast to a recent study of Indiana’s section 1115 Medicaid waiver program that found the waiver was associated with lower Medicaid enrollment compared to traditional Medicaid expansion states [2]. The differences in findings between Indiana and Arkansas are likely related to differences in timing and implementation of Medicaid expansion [9–11]. Arkansas expanded in 2014 and enrolled beneficiaries in the “private option,” which is the name given to the premium assistance to buy private insurance in the health insurance exchanges, regardless of income level [9]. Arkansas’ waiver also created health savings accounts that required contributions by beneficiaries to avoid out-of-pocket cost sharing, but this program was not fully implemented and ultimately eliminated in 2016 [9]. Indiana expanded Medicaid in 2015 as an extension of a pre-ACA waiver using a health savings account that, unlike Arkansas, was fully implemented and levied a penalty on beneficiaries that failed to contribute to the account, and also included a financial incentive for meeting healthy behavior criteria [9]. Moreover, Indiana’s waiver penalized beneficiaries for non-emergency use of a hospital emergency department [9]. Therefore, we speculate that the primary mechanisms for the difference in Medicaid enrollment between the two states are likely that Indiana already had a similar waiver in place prior to the ACA, Arkansas’ waiver automatically enrolled participants in premium assistance for the health insurance exchange, and Indiana has more penalties in place than Arkansas for beneficiaries [2, 10–11].

Our finding is consistent with other studies that found Medicaid expansion is associated with improved access to care [6–7, 10–11]. Evaluation of Medicaid waivers will continue to be important as states propose alternatives to conventional Medicaid policy [3, 9]. Arkansas was granted approval by CMS to add work requirements to their Section 1115 waiver in 2018 [4]. However, a federal judge threw out the work requirement provision of the waiver, which led CMS to direct Arkansas to end the work requirement provision [4]. The current dynamic policy environment for experimentation with waivers driven by political directives and judicial oversight will motivate the need for continued study of Medicaid waivers.
